# Admission METS-IR in relation to severe acute pancreatitis among patients with hypertriglyceridemic acute pancreatitis: a retrospective cohort study

**DOI:** 10.3389/fmed.2026.1999625

**Published:** 2026-10-15

**Authors:** Ziyi Zhang, Changwei Peng, Hong Peng, Yuanxia Zhu, Chenghu Huang, Li Rong

**Affiliations:** 1 Department of Gastroenterology, Bishan Hospital of Chongqing Medical University, Chongqing, China; 2 Department of Endocrinology, Bishan Hospital of Chongqing Medical University, Chongqing, China; 3 Department of Gastroenterology, Chongqing Jiulongpo People’s Hospital, Jiulongpo Hospital of Chongqing, Chongqing, China

**Keywords:** hypertriglyceridemic acute pancreatitis, insulin resistance, METS-IR, retrospective cohort study, severe acute pancreatitis

## Abstract

**Background:**

Hypertriglyceridemic acute pancreatitis (HTG-AP) is accompanied by significant metabolic abnormalities, but few studies have investigated whether a composite measure of this metabolic burden is associated with severe acute pancreatitis (SAP). The metabolic score for insulin resistance (METS-IR), derived from fasting glucose, high-density lipoprotein cholesterol, triglycerides, and body mass index, can be calculated from routinely available clinical data without requiring an insulin assay. However, its association with SAP in HTG-AP remains poorly characterized.

**Objective:**

This study investigates the association between admission METS-IR and SAP in patients with HTG-AP and assesses the robustness of this association through subgroup and sensitivity analyses.

**Methods:**

We retrospectively assembled a single-center cohort of 456 adults admitted with HTG-AP between January 2019 and July 2025. Logistic regression evaluated METS-IR as a continuous exposure and in quartiles. Restricted cubic splines, subgroup analyses, and sensitivity analyses were performed. Exploratory ROC analyses compared apparent discrimination with TyG, TyG-BMI, and BISAP.

**Results:**

SAP occurred in 78 of 456 patients (17.1%). Median METS-IR was 69.99 (IQR, 61.92–83.67) in the SAP group and 58.18 (IQR, 51.47–66.46) in the non-SAP group. In Model 3, each one-point increase in METS-IR was associated with higher odds of SAP (OR, 1.06; 95% CI, 1.04–1.08). After additional adjustment for TG, TG/HDL-C, and TyG, the estimate was attenuated (OR, 1.03; 95% CI, 0.997–1.064; *P* = 0.073). Apparent AUCs were 0.730 for METS-IR, 0.720 for TyG, and 0.758 for BISAP; neither paired comparison showed a statistically significant difference. In the three-category analysis, the METS-IR association was stronger for SAP vs. MAP than for MSAP vs. MAP.

**Conclusion:**

Higher admission METS-IR was independently associated with higher odds of SAP in patients with HTG-AP. METS-IR may provide a readily available measure of metabolic burden for early clinical assessment of patients with HTG-AP, although its clinical utility requires confirmation in prospective multicenter studies.

## 1 Introduction

Acute pancreatitis (AP) has a heterogeneous clinical course: most episodes resolve without persistent organ failure, whereas a subset progresses to severe acute pancreatitis (SAP) (1, 2). Hypertriglyceridemia-associated acute pancreatitis (HTG-AP) is a clinically important subtype. Recent evidence documents its burden of severe disease and recurrence (3, 4). In a separate cohort restricted to mild HTG-AP, insulin-resistance-related indices, including METS-IR, were associated with recurrence; that study did not address SAP during the index admission (5). Importantly, admission triglyceride concentration alone does not fully account for severity in HTG-AP (4). Current AP guidelines recommend early assessment of severity (6), and contemporary HTG-AP studies have examined BISAP alongside biochemical and metabolic measures (7–9). These findings motivate an examination of the association between an admission composite metabolic measure and SAP, with BISAP used as a descriptive comparator.

The metabolic phenotype of HTG-AP provides a rationale for this question. In HTG-AP and general AP cohorts, individual metabolic syndrome components have been associated with adverse outcomes (10, 11). In obesity, impaired insulin suppression of adipose lipolysis increases circulating free fatty acids; low-grade adipose inflammation can accompany insulin resistance, although its causal contribution remains debated (12). These changes may contribute to dyslipidemia through increased fatty-acid delivery and altered hepatic triglyceride-rich lipoprotein metabolism (13). In HTG-AP, lipolysis of circulating triglycerides can generate non-esterified fatty acids; mechanistic and translational evidence links this process to pancreatic and systemic injury (14–17). In a general AP cohort, HOMA-estimated insulin resistance was also associated with adverse outcomes (18). These observations suggest biological plausibility, but they do not establish that pre-existing insulin resistance causes SAP in our cohort.

The metabolic score for insulin resistance (METS-IR) combines fasting glucose, triglycerides, high-density lipoprotein cholesterol (HDL-C), and body mass index (BMI). It was developed as a non-insulin-based surrogate of insulin sensitivity outside acute illness (19). During AP, its components may change even before treatment; an admission value should therefore be interpreted as an acute-phase metabolic measure rather than a direct estimate of pre-illness insulin resistance. Kou et al. recently studied METS-IR with BISAP in a retrospective model for severe hyperlipidemic AP (20). Other recent HTG-AP studies associated TyG or related metabolic indices with SAP or severe progression (21–23), whereas a study of TyG-BMI in critically ill AP assessed mortality, a different outcome (24). The present analysis focuses on the association between METS-IR and SAP in HTG-AP.

Therefore, this study investigated admission METS-IR as the primary exposure in patients with HTG-AP. It assessed its adjusted association with SAP and examined the potential shape and nonlinearity of this relationship. It further evaluated the robustness and consistency of the findings through subgroup and sensitivity analyses. Comparisons with the triglyceride-glucose (TyG) index, TyG-BMI, and BISAP were exploratory.

## 2 Materials and methods

### 2.1 Study participants

Consecutive adult admissions to the gastroenterology and critical care departments of Bishan Hospital, Chongqing Medical University, with HTG-AP between January 2019 and July 2025, were retrospectively reviewed. The final cohort comprised 456 patients who met all eligibility criteria. Patients were included if they fulfilled all of the following criteria: (1) a diagnosis of AP based on the revised Atlanta criteria, defined by the presence of at least two of three features: characteristic abdominal pain, serum amylase and/or lipase levels greater than three times the upper limit of normal, and imaging findings compatible with AP (25); (2) a serum TG concentration ≥11.3 mmol/L, or a concentration of 5.65–11.3 mmol/L accompanied by lactescent serum after other established etiologies had been excluded; (3) age ≥18 years; and (4) complete clinical data available for analysis. Patients were excluded if they met any of the following criteria: (1) AP predominantly due to other etiologies, including biliary, alcoholic, drug-induced, traumatic, postoperative, or other causes; (2) concomitant malignancy, pregnancy, or lactation; (3) time from symptom onset to admission >72 h; or (4) a history of chronic pancreatitis. The study selection process is presented in Figure 1. Figure 1 shows that 411 of the 867 records remaining after the other listed exclusions (47.4%) were excluded for incomplete clinical data. All 411 records excluded at this stage lacked at least one valid pretreatment fasting measurement or confirmation of fasting status required to calculate METS-IR. Because the exposure could not be defined reliably for those records, the primary analysis used complete cases. The retained 456-person analytic file does not establish variable-specific missingness among excluded records.

**Figure 1:**
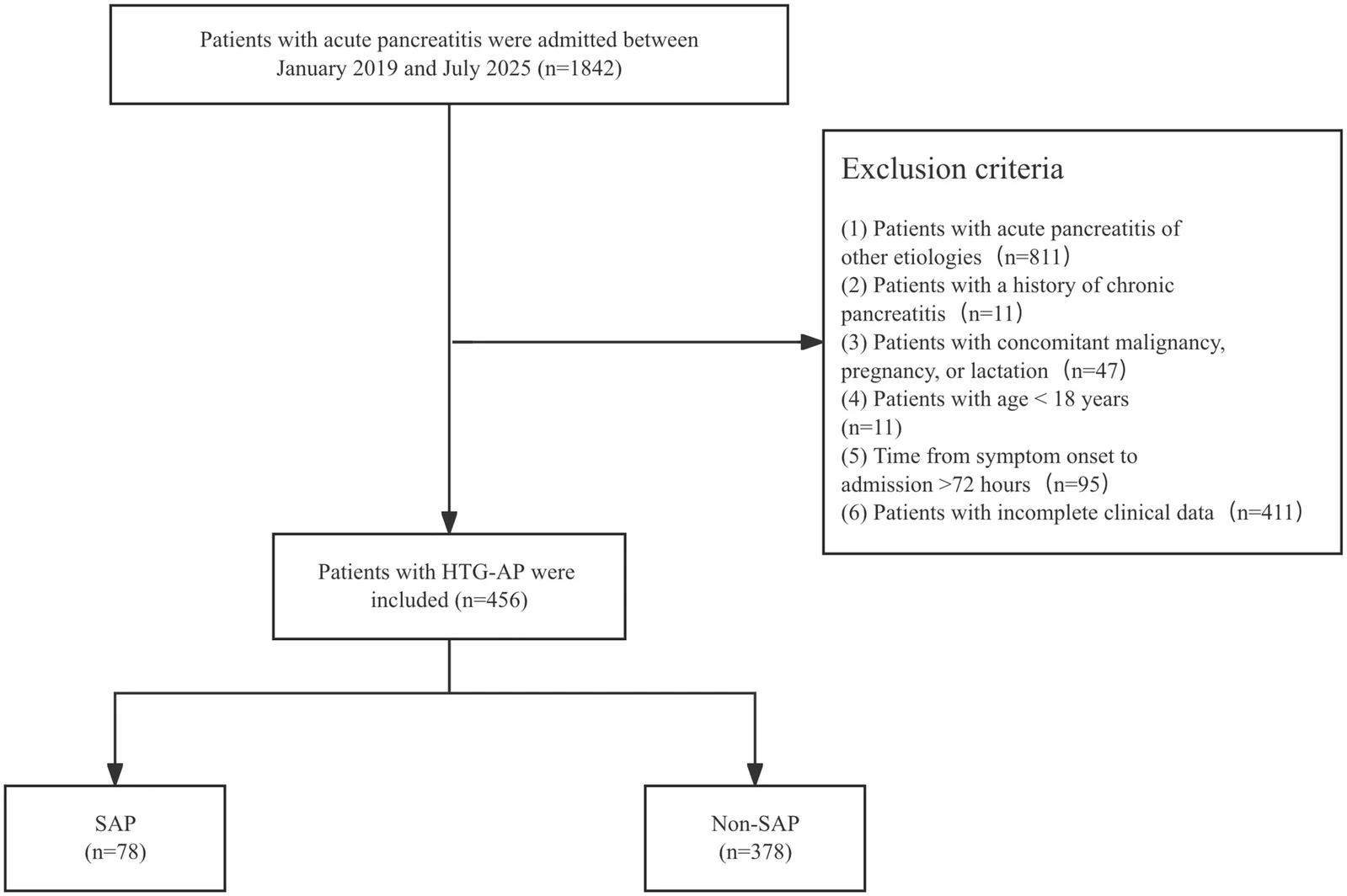
Flowchart of the study.

The study protocol was approved by the Medical Ethics Committee of Bishan Hospital of Chongqing Medical University (No. cqbyky11-20250926-01). The requirement for written informed consent was waived because this retrospective study used anonymized clinical data. All study procedures were conducted in accordance with the ethical principles of the Declaration of Helsinki and its subsequent amendments.

### 2.2 Data collection

Clinical variables recorded during the first 24 h of hospitalization were retrospectively extracted. These included demographic characteristics, lifestyle factors, anthropometric measurements, comorbidities, inflammatory markers, electrolytes, blood counts, fasting glucose, renal and liver indices, and lipid measurements. The fasting glucose, TG, and HDL-C values used to calculate METS-IR came from fasting venous blood collected before the initiation of acute treatment in every included patient. For patients who were not fasting on arrival, the selected sample was obtained after 6–8 h without oral intake and before treatment. Patients whose relevant blood samples were collected after treatment initiation were excluded during screening, as reflected in Figure 1; the final analytic cohort remained 456. Although the order of sampling and treatment was verified, the exact elapsed time from blood collection to first acute treatment could not be reconstructed consistently from the retrospective records. The exact interval from symptom onset to sampling was not available for all patients. According to the reviewed pre-admission records, no included patient had received glucose-lowering or lipid-lowering treatment before admission. TyG, TyG-BMI, and METS-IR were calculated as follows (19, 23, 24):

$$\mathrm{TyG}\;=\;\ln\left[\mathrm{FPG}\left(\frac{\mathrm{mg}}{\mathrm{dL}}\right)\;\times \;\mathrm{TG}\;\left(\frac{\mathrm{mg}}{\mathrm{dL}}\right)/2\right]$$

$$\mathrm{TyG}-\mathrm{BMI}\;=\;\mathrm{TyG}\;\times \;\mathrm{BMI}\;\left(\frac{\mathrm{kg}}{m^{2}}\right)$$

$$\mathrm{METS}-\mathrm{IR}\;=\;\ln\left[2\;\times \;\mathrm{FBG}\;\left(\frac{\mathrm{mg}}{\mathrm{dL}}\right)\;+\;\mathrm{TG}\;\left(\frac{\mathrm{mg}}{\mathrm{dL}}\right)\right]\;\times \;\mathrm{BMI}\;\left(\frac{\mathrm{kg}}{m^{2}}\right)/\ln\left[\mathrm{HDL}-C\;\left(\frac{\mathrm{mg}}{\mathrm{dL}}\right)\right]$$

### 2.3 Outcome definition

According to the 2012 revised Atlanta classification (25), AP severity was categorized as mild acute pancreatitis (MAP), moderately severe acute pancreatitis (MSAP), or SAP. MAP was defined as the absence of organ failure and local or systemic complications. MSAP was defined by transient organ failure lasting ≤48 h and/or local or systemic complications, whereas SAP was defined by persistent organ failure lasting >48 h. For the primary analysis, patients with MAP (*n* = 245) and MSAP (*n* = 133) were combined into the non-SAP group.

### 2.4 Statistical analysis

All analyses were performed in R 4.6.1. Continuous variables were summarized as medians and interquartile ranges and compared using the Wilcoxon rank-sum test. Categorical variables were summarized as *n* (%) and compared using Pearson's chi-square or Fisher's exact test, as appropriate. Logistic regression evaluated METS-IR continuously and by quartile. Model 1 was unadjusted; Model 2 adjusted for age and sex; Model 3 additionally adjusted for hypertension, diabetes, fatty liver disease, smoking, and alcohol use. Age, sex, smoking, and alcohol exposure were selected as plausible pre-admission common causes of metabolic status and AP severity. Documented hypertension, diabetes, and fatty liver disease were included as pre-existing metabolic characteristics associated with both variables, informed by prior AP studies (10, 11, 26, 27); they may also reflect long-standing metabolic dysfunction and are not proven confounders. Model 3 therefore estimates a conditional association, not a causal effect. An expanded sensitivity model also included WBC, CRP, calcium, and creatinine. Because these were measured during AP and may reflect inflammation, organ dysfunction, or pathways to severity, the expanded model has a different interpretation and was not treated as adjustment for baseline confounding. Restricted cubic splines, subgroup analyses, and the analysis excluding diabetes followed the approach described above.

Additional exploratory analyses sequentially added TG, the TG/HDL-C ratio, and TyG to Model 3 on the same complete-case sample. Paired DeLong tests compared the AUCs of METS-IR with TyG and BISAP on matched participants. A multinomial model used MAP as the reference category to compare MSAP and SAP. A five-fold cross-validated LASSO logistic model at lambda.1se examined selection among METS-IR and Model 3 covariates; selection was not used for causal inference. No imputation was performed for missing or unverifiable fasting glucose and TG measurements used to define the exposure.

## 3 Results

### 3.1 Baseline characteristics

The study cohort comprised 456 patients, of whom 378 (82.9%) were classified as having non-SAP and 78 (17.1%) as having SAP. The median age was 43.50 years (IQR, 36.00–51.00 years). No significant differences were observed between the two groups in age, sex, BMI, marital status, alcohol consumption, smoking, hypertension, or diabetes (all *P* > 0.05). Compared with patients in the non-SAP group, those with SAP had significantly higher WBC and neutrophil counts, mean platelet volume (MPV), platelet distribution width (PDW), fasting glucose, TG, LDL-C, total cholesterol, AST, TyG, and TyG-BMI levels. Lymphocyte and eosinophil counts, sodium, calcium, HDL-C, and creatinine levels were significantly lower in the SAP group (all *P* < 0.05). Fatty liver disease and systemic inflammatory response syndrome (SIRS) were also more frequently observed among patients with SAP. Admission METS-IR differed significantly between groups, with higher values in patients who developed SAP than in those with non-SAP [69.99 (IQR, 61.92–83.67) vs. 58.18 (IQR, 51.47–66.46), respectively; *P* < 0.001] (Table 1).

**Table 1:** Baseline characteristics of patients with hypertriglyceridemic acute pancreatitis according to disease severity.

Characteristic	Overall (*N* = 456)	Non-SAP (*N* = 378)	SAP (*N* = 78)	*P* value
Age, years	43.50 (36.00–51.00)	43.50 (36.00–51.00)	43.50 (35.25–50.00)	0.884
Sex, *n* (%)				0.833
Female	130 (28.5%)	107 (28.3%)	23 (29.5%)	
Male	326 (71.5%)	271 (71.7%)	55 (70.5%)	
BMI, kg/m^2^	26.63 (24.28–29.05)	26.48 (24.26–28.66)	27.06 (24.85–30.03)	0.109
WBC, 10^9^/L	14.22 (11.13–17.14)	13.94 (11.07–16.88)	15.16 (12.56–18.30)	0.007
Neutrophil, 10^9^/L	11.66 (8.78–14.57)	11.29 (8.60–14.25)	13.06 (10.41–15.88)	0.001
Monocyte, 10^9^/L	0.59 (0.36–0.83)	0.58 (0.36–0.80)	0.64 (0.42–1.19)	0.076
CRP, mg/L	9.87 (3.03–41.99)	9.59 (2.65–40.63)	11.41 (5.20–47.37)	0.174
Lymphocyte, 10^9^/L	1.52 (0.92–2.17)	1.56 (0.99–2.21)	1.23 (0.73–1.97)	0.024
Erythrocyte, 10^12^/L	4.91 (4.54–5.32)	4.92 (4.53–5.33)	4.76 (4.55–5.29)	0.392
HCT, %	44.60 (40.60–47.32)	44.75 (40.62–47.38)	43.75 (40.62–47.00)	0.676
MPV, fL	10.65 (9.70–11.90)	10.50 (9.60–11.60)	11.65 (10.20–12.38)	<0.001
PDW, fL	16.90 (16.50–17.20)	16.80 (16.50–17.10)	17.00 (16.80–17.30)	0.001
Platelet, 10^9^/L	207.00 (167.00–257.00)	212.00 (169.00–257.75)	187.00 (157.50–241.25)	0.057
Hemoglobin, g/L	159.00 (146.00–170.00)	159.00 (145.00–169.00)	161.00 (147.00–177.75)	0.168
Eosinophil, 10^9^/L	0.06 (0.02–0.12)	0.07 (0.02–0.13)	0.04 (0.01–0.07)	<0.001
Basophil, 10^9^/L	0.02 (0.01–0.04)	0.02 (0.01–0.04)	0.03 (0.02–0.04)	0.180
Sodium, mmol/L	132.00 (129.00–134.00)	132.00 (129.00–134.00)	130.50 (128.00–133.75)	0.006
Potassium, mmol/L	3.87 (3.63–4.19)	3.88 (3.66–4.20)	3.84 (3.55–4.13)	0.299
Calcium, mmol/L	2.27 (2.15–2.38)	2.28 (2.17–2.38)	2.16 (1.95–2.33)	<0.001
FBG, mmol/L	10.20 (7.21–14.44)	9.70 (7.00–14.13)	11.52 (8.54–15.60)	0.003
Triglycerides, mmol/L	12.41 (7.42–18.48)	10.96 (7.06–17.14)	18.18 (13.14–21.24)	<0.001
HDL-C, mmol/L	0.65 (0.50–0.85)	0.68 (0.53–0.87)	0.46 (0.37–0.59)	<0.001
LDL-C, mmol/L	1.96 (1.35–3.39)	1.90 (1.32–2.86)	3.67 (1.59–5.71)	<0.001
Total cholesterol, mmol/L	7.44 (5.75–10.29)	7.13 (5.59–9.91)	9.31 (6.79–11.80)	<0.001
Urea nitrogen, mmol/L	4.34 (3.31–5.53)	4.35 (3.31–5.53)	4.24 (3.50–5.52)	0.788
Creatinine, μmol/L	54.00 (44.00–64.00)	54.00 (45.00–64.00)	47.00 (40.25–62.00)	0.025
ALT, U/L	27.00 (18.75–40.00)	27.00 (19.00–39.00)	27.00 (16.50–40.75)	0.682
AST, U/L	27.00 (20.00–36.00)	26.00 (20.00–35.00)	32.50 (25.00–41.75)	<0.001
Albumin, g/L	41.45 (37.91–43.98)	41.55 (38.06–43.99)	41.34 (37.39–43.92)	0.696
METS-IR	59.54 (51.85–69.11)	58.18 (51.47–66.46)	69.99 (61.92–83.67)	<0.001
TyG	11.48 (10.89–11.99)	11.37 (10.78–11.87)	11.94 (11.58–12.34)	<0.001
TyG-BMI	303.09 (274.36–335.51)	300.18 (272.89–330.57)	329.11 (287.15–362.06)	<0.001
BISAP	1.00 (0.00–1.00)	1.00 (0.00–1.00)	1.00 (1.00–2.00)	<0.001
Diabetes, *n* (%)				0.108
No	321 (70.4%)	272 (72.0%)	49 (62.8%)	
Yes	135 (29.6%)	106 (28.0%)	29 (37.2%)	
Marital status, *n* (%)				0.608
Unmarried	67 (14.7%)	57 (15.1%)	10 (12.8%)	
Married	389 (85.3%)	321 (84.9%)	68 (87.2%)	
Alcohol consumption, *n* (%)				0.221
No	302 (66.2%)	255 (67.5%)	47 (60.3%)	
Yes	154 (33.8%)	123 (32.5%)	31 (39.7%)	
Smoking, *n* (%)				0.348
No	324 (71.1%)	272 (72.0%)	52 (66.7%)	
Yes	132 (28.9%)	106 (28.0%)	26 (33.3%)	
Hypertension, *n* (%)				0.103
No	364 (79.8%)	307 (81.2%)	57 (73.1%)	
Yes	92 (20.2%)	71 (18.8%)	21 (26.9%)	
Fatty liver, *n* (%)				<0.001
No	224 (49.1%)	202 (53.4%)	22 (28.2%)	
Yes	232 (50.9%)	176 (46.6%)	56 (71.8%)	
SIRS, *n* (%)				<0.001
No	230 (50.4%)	206 (54.5%)	24 (30.8%)	
Yes	226 (49.6%)	172 (45.5%)	54 (69.2%)	

Values are presented as median (interquartile range) for continuous variables and *n* (%) for categorical variables. ALT, alanine aminotransferase; AST, aspartate aminotransferase; BMI, body mass index; CRP, C-reactive protein; FBG, fasting blood glucose; HCT, hematocrit; HDL-C, high-density lipoprotein cholesterol; LDL-C, low-density lipoprotein cholesterol; METS-IR, metabolic score for insulin resistance; MPV, mean platelet volume; Non-SAP, non-severe acute pancreatitis; PDW, platelet distribution width; SAP, severe acute pancreatitis; SIRS, systemic inflammatory response syndrome; TyG, triglyceride-glucose index; TyG-BMI, triglyceride-glucose index multiplied by body mass index; WBC, white blood cell count.

### 3.2 Association between METS-IR and SAP

Across Models 1–3, each one-point increase in METS-IR was associated with higher odds of SAP. The corresponding ORs were 1.06 (95% CI, 1.04–1.08), 1.07 (95% CI, 1.05–1.09), and 1.06 (95% CI, 1.04–1.08), respectively (all *P* < 0.001). With Q1 as the reference category, Model 3 yielded ORs of 0.62 (95% CI, 0.22–1.74) for Q2, 2.11 (95% CI, 0.90–4.95) for Q3, and 5.81 (95% CI, 2.53–13.34) for Q4. Only Q4 differed significantly from Q1, whereas the ordered trend across quartiles was significant in all three models (Table 2).

**Table 2:** Association between METS-IR and the odds of SAP.

Characteristic	Model 1		Model 2		Model 3	
	OR (95% CI)	*P* value	OR (95% CI)	*P* value	OR (95% CI)	*P* value
METS-IR (Continuous)	1.06 (1.04–1.08)	<0.001	1.07 (1.05–1.09)	<0.001	1.06 (1.04–1.08)	<0.001
METS-IR quartiles						
Q1	Reference		Reference		Reference	
Q2	0.68 (0.25–1.85)	0.452	0.72 (0.26–1.98)	0.527	0.62 (0.22–1.74)	0.366
Q3	2.08 (0.92–4.70)	0.078	2.45 (1.06–5.65)	0.036	2.11 (0.90–4.95)	0.087
Q4	6.07 (2.86–12.87)	<0.001	7.57 (3.42–16.78)	<0.001	5.81 (2.53–13.34)	<0.001
*P* for trend		<0.001		<0.001		<0.001

Model 1 was unadjusted. Model 2 was adjusted for age and sex. Model 3 was further adjusted for hypertension, diabetes, fatty liver disease, smoking, and alcohol consumption. Quartile boundaries were as follows: Q1 ≤ 51.85; Q2 > 51.85 to ≤59.54; Q3 > 59.54 to ≤69.11; and Q4 > 69.11. CI, confidence interval; OR, odds ratio; Q, quartile; METS-IR, metabolic score for insulin resistance; SAP, severe acute pancreatitis.

Sequential adjustment on the same 456 patients attenuated the METS-IR estimate from OR 1.061 (95% CI, 1.041–1.082) in Model 3 to 1.055 (1.035–1.076) after TG, 1.041 (1.009–1.073) after TG and TG/HDL-C, and 1.030 (0.997–1.064; *P* = 0.073) after additionally including TyG (Supplementary Table 3 and Supplementary Figure 1). The overlapping composition of these indices limits interpretation of the fully adjusted coefficient.

### 3.3 Dose-response relationship between METS-IR and SAP

The restricted cubic spline analysis showed a significant overall association between METS-IR and SAP (*P* < 0.001), whereas the test for nonlinearity was not significant (*P* = 0.138). The estimated association declined slightly at lower METS-IR values, where the confidence interval was relatively wide, and then increased across higher METS-IR values (Figure 2).

**Figure 2:**
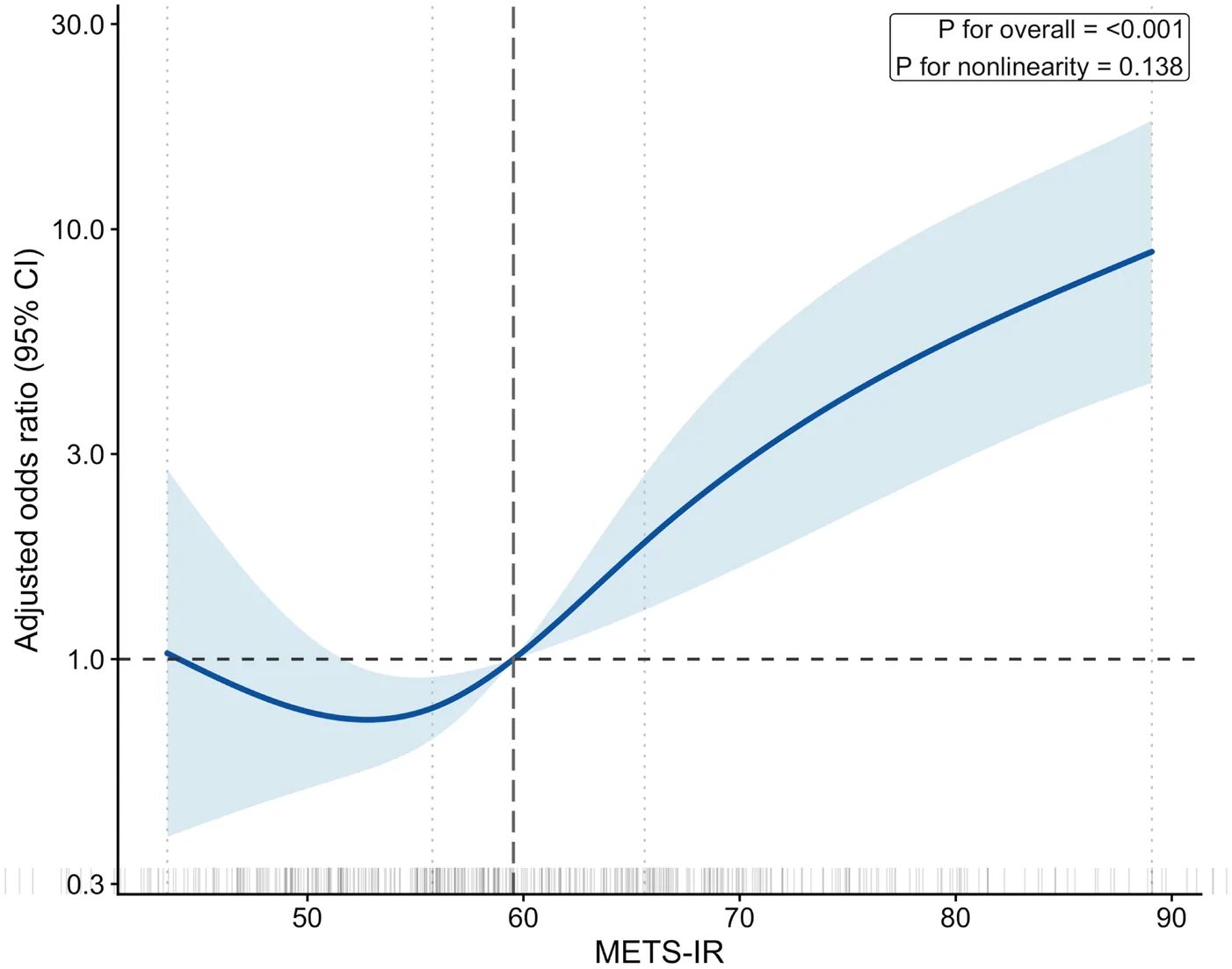
Adjusted spline association between admission metabolic score for insulin resistance (METS-IR) and the odds of severe acute pancreatitis. The solid curve represents the adjusted odds ratio (OR), and the shaded band represents the corresponding 95% confidence interval (CI). The vertical dashed line indicates the reference value of 59.54. Estimates were adjusted for age, sex, hypertension, diabetes, fatty liver disease, smoking, and alcohol consumption.

### 3.4 Subgroup analyses

Stratified analyses were performed according to age, sex, diabetes, hypertension, fatty liver disease, smoking status, and alcohol consumption. The adjusted estimates were positive across all examined strata. None of the interaction tests reached statistical significance, indicating no evidence that the association between METS-IR and SAP differed according to the prespecified subgroup factors (Figure 3).

**Figure 3:**
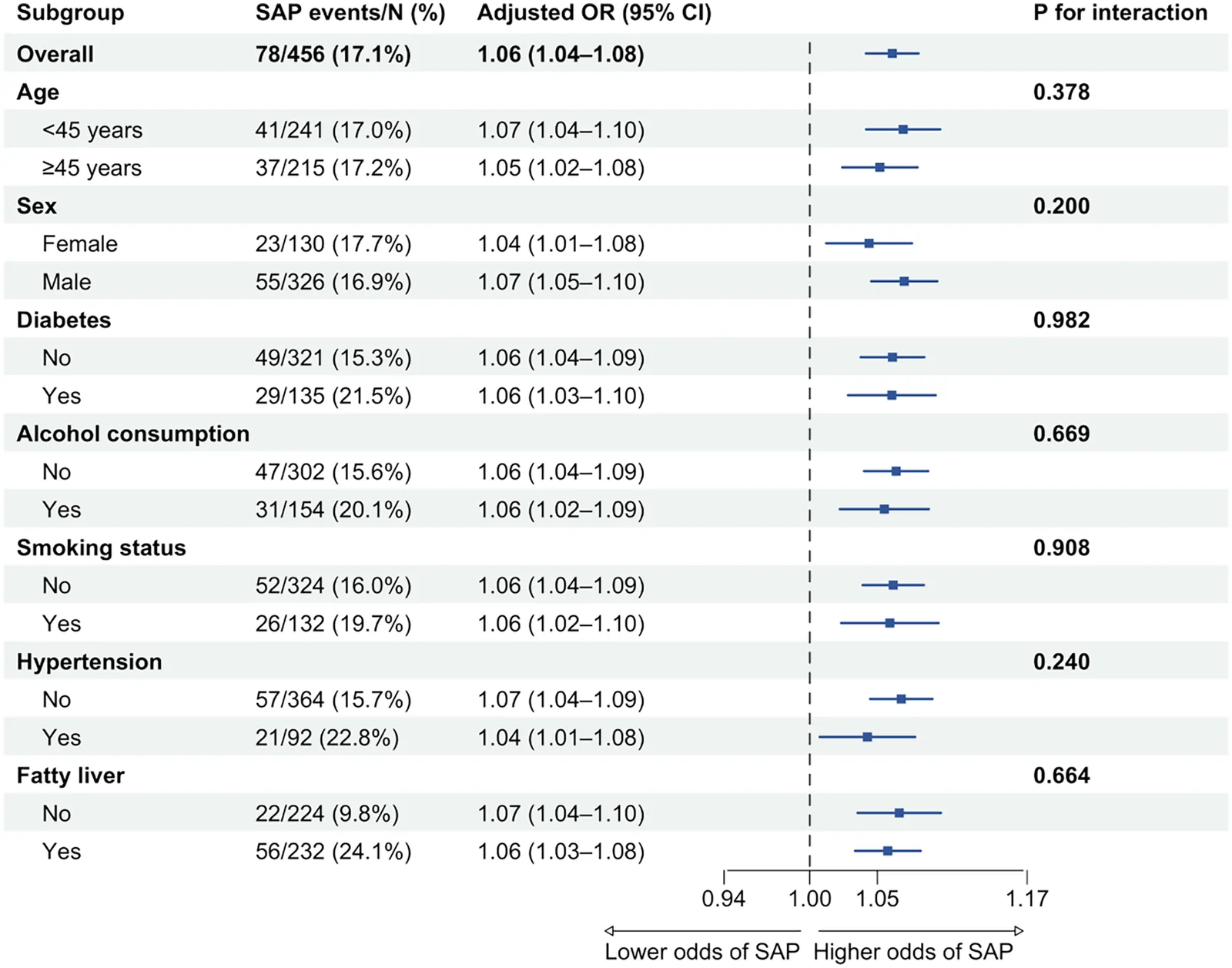
Subgroup analyses of the association between admission metabolic score for insulin resistance (METS-IR) and severe acute pancreatitis.

### 3.5 Discriminatory ability

In the exploratory ROC analysis, METS-IR showed an apparent AUC of 0.730 (95% CI, 0.663–0.798) for discriminating SAP from non-SAP. The corresponding AUCs were 0.720 (95% CI, 0.661–0.778) for TyG and 0.644 (95% CI, 0.572–0.715) for TyG-BMI (Figure 4).

**Figure 4:**
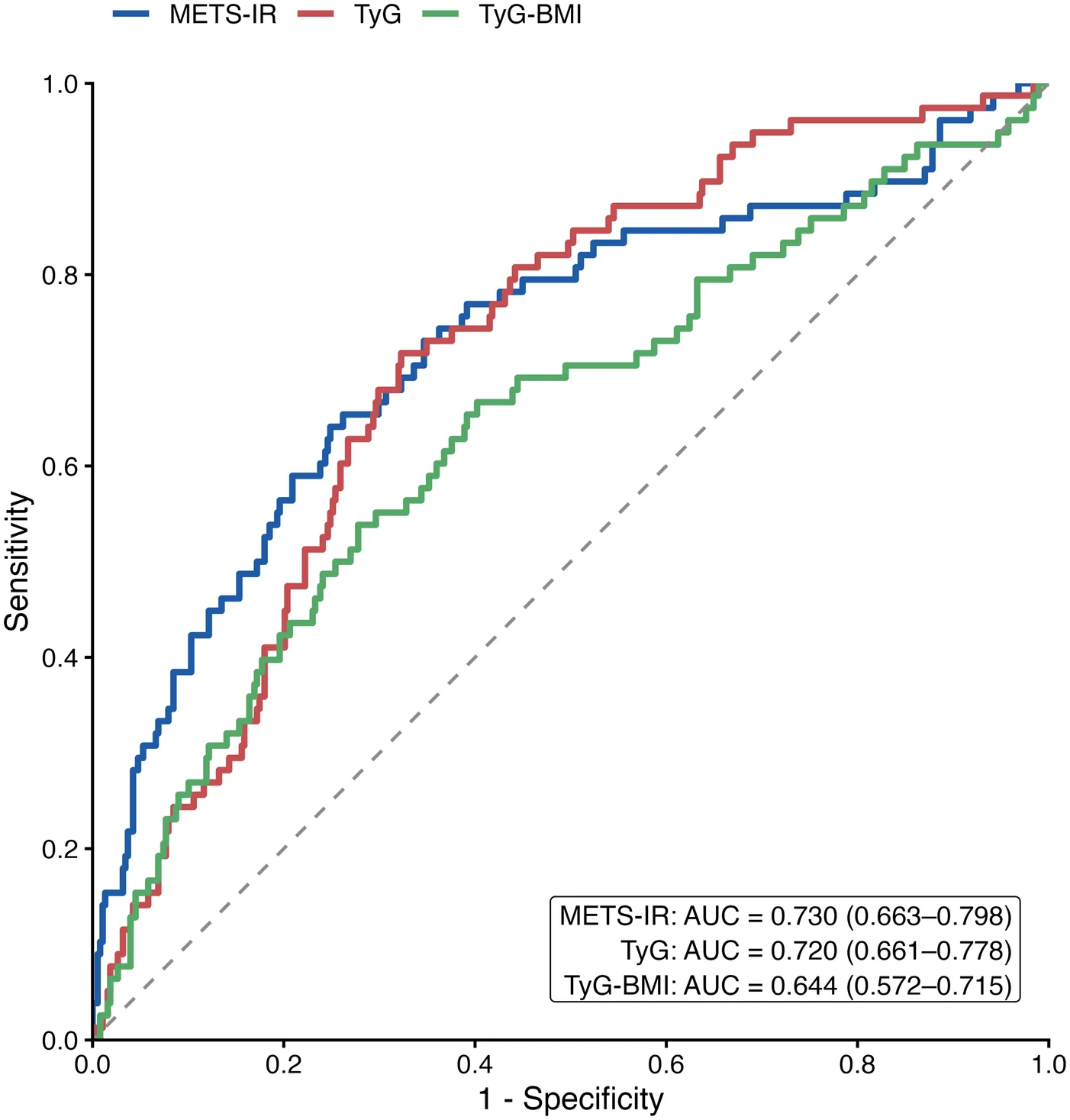
Receiver operating characteristic curves for METS-IR, TyG, and TyG-BMI in predicting severe acute pancreatitis. AUC, area under the curve; METS-IR, metabolic score for insulin resistance; TyG, triglyceride-glucose index; TyG-BMI, triglyceride-glucose index multiplied by body mass index.

The paired METS-IR vs. TyG AUC difference was 0.011 (DeLong *P* = 0.737). On the same 456 patients, BISAP had an AUC of 0.758 (95% CI, 0.703–0.812), compared with 0.730 for METS-IR (paired DeLong *P* = 0.521; Figure 5 and Supplementary Table 4). Neither comparison established superiority or equivalence.

**Figure 5:**
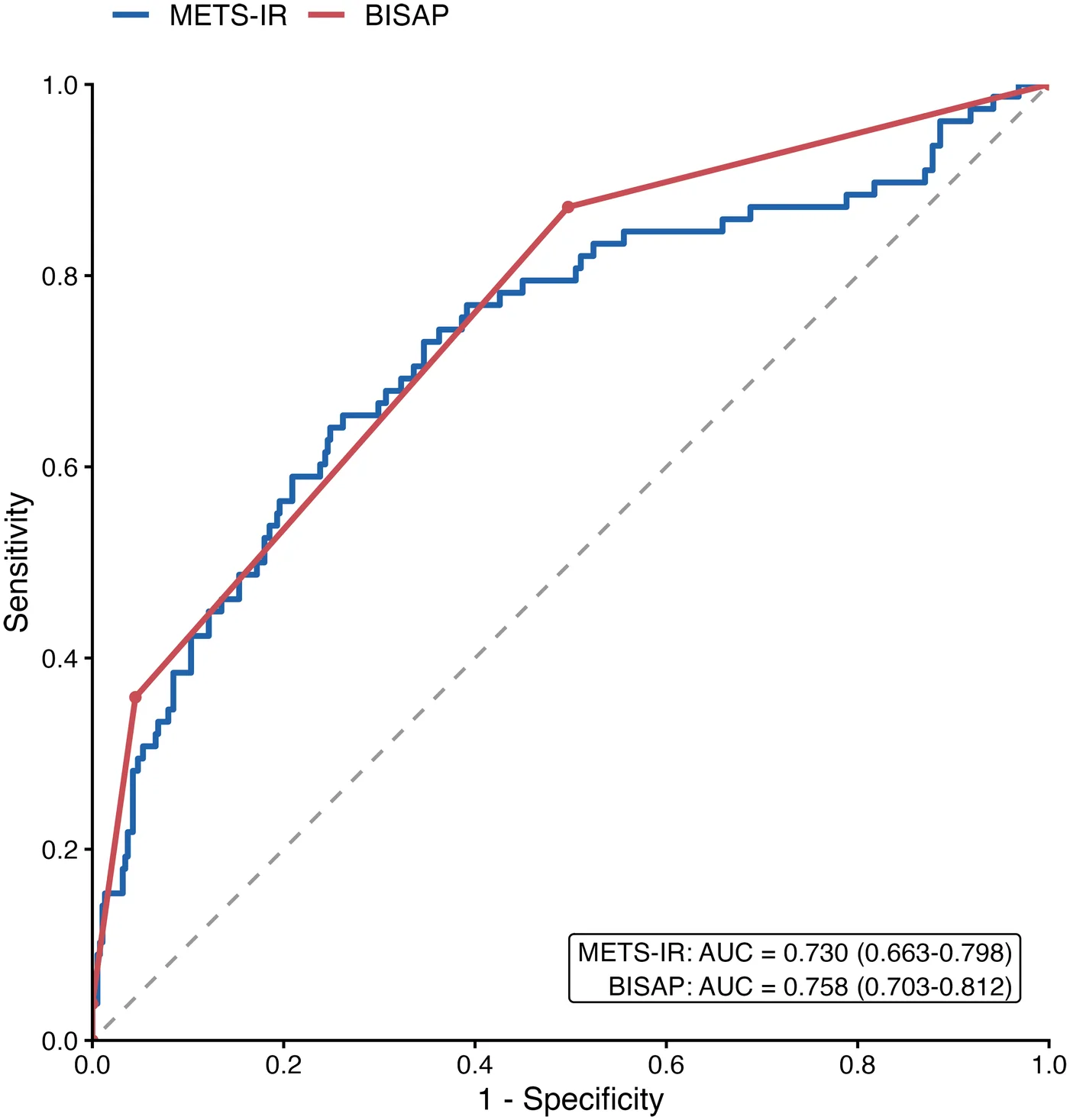
Exploratory ROC comparison of admission METS-IR and BISAP for SAP in the same 456 patients.

### 3.6 Sensitivity analysis

After excluding 135 patients with diabetes, 321 patients remained, including 49 with SAP. Using the quartile boundaries derived from the main cohort, Model 3 yielded an OR of 1.06 (95% CI, 1.04–1.09) per one-point increase in METS-IR and an OR of 7.12 (95% CI, 2.58–22.45) for Q4 vs. Q1. The ordered trend across quartiles remained significant (Supplementary Table 1). The expanded sensitivity model included all 456 patients. After adding WBC, CRP, calcium, and creatinine to Model 3, the estimates changed little. The OR per one-point increase in METS-IR was 1.06 (95% CI, 1.04–1.08), while the OR for Q4 vs. Q1 was 6.25 (95% CI, 2.56–15.28). The quartile trend remained significant (Supplementary Table 2).

In the three-category sensitivity analysis with MAP as reference, the OR per one-point increase in METS-IR was 1.019 (95% CI, 1.001–1.039) for MSAP vs. MAP and 1.070 (1.048–1.093) for SAP vs. MAP (Supplementary Table 5 and Supplementary Figure 3). In an exploratory LASSO model containing METS-IR and Model 3 covariates, only METS-IR retained a nonzero coefficient at lambda.1se (Supplementary Table 6 and Supplementary Figures 4, 5). This selection result does not establish independence from the metabolic indices absent from that candidate set.

## 4 Discussion

In this single-center cohort of patients with hypertriglyceridemia-associated acute pancreatitis (HTG-AP), higher admission METS-IR was associated with severe acute pancreatitis (SAP) after adjustment for selected demographic, lifestyle, and pre-existing metabolic characteristics. In the three-category sensitivity analysis, the association was stronger for SAP than for moderately severe acute pancreatitis when mild acute pancreatitis was the reference. Together, these findings identify an admission metabolic profile associated with disease severity. They do not establish that pre-existing insulin resistance preceded or caused SAP.

The association is biologically plausible in the metabolic context of HTG-AP. Excess triglycerides provide substrate for lipolysis, and mechanistic and translational studies indicate that the resulting non-esterified fatty acids can aggravate pancreatic injury, systemic inflammation, and organ dysfunction (14–17). Clinical studies have also associated metabolic syndrome components with adverse outcomes in AP, including HTG-AP (10, 11). METS-IR combines fasting glucose, triglycerides, HDL-C, and body mass index, and was originally developed as a surrogate of insulin sensitivity in a non-acute setting (19). It may therefore summarize several aspects of the metabolic state relevant to HTG-AP. Nevertheless, our study did not directly measure insulin sensitivity, fatty-acid generation, or inflammatory pathways. The proposed mechanism remains an interpretation rather than a demonstrated explanation of the observed association.

Previous studies have associated HOMA-estimated insulin resistance and the triglyceride-glucose (TyG) index with AP severity (18, 21–23). More directly, Kou et al. studied METS-IR and BISAP in a model for severe hyperlipidemic AP (20). Their reported METS-IR AUCs of 0.743 in the training set and 0.719 in the internal validation set were similar in magnitude to the apparent AUC of 0.730 in our cohort. Their BISAP AUCs of 0.870 and 0.865 were higher than the 0.758 observed here. Differences in patient selection, etiologic definitions, disease spectrum, and score distributions may contribute to this discrepancy; AUCs from separate cohorts cannot be compared as though they arose from a paired experiment. In our paired analyses, the apparent AUC of METS-IR did not differ significantly from those of TyG or BISAP. These findings establish neither equivalent performance nor an incremental contribution beyond an established severity score.

The overlap among metabolic indices is also important for interpreting the adjusted association. Fasting glucose, triglycerides, and HDL-C differed between the SAP and non-SAP groups, whereas body mass index did not differ significantly. This pattern alone cannot determine the contribution of each METS-IR component. In sequential models, additional adjustment for triglycerides and the triglyceride-to-HDL-C ratio attenuated the association; after further adjustment for TyG, the METS-IR estimate was 1.030 per point (95% CI, 0.997–1.064; *P* = 0.073). Because these indices share measured components, simultaneous adjustment introduces mathematical overlap and potential collinearity. The attenuation indicates substantial shared information but does not isolate a causal component of METS-IR or prove that its other components are uninformative.

The covariate models answer related but distinct questions. Model 3 adjusted for characteristics known before the acute episode, including age, sex, lifestyle factors, and documented metabolic comorbidities. However, diabetes, hypertension, and fatty liver disease may themselves reflect longstanding metabolic dysfunction rather than function solely as confounders. The expanded model additionally conditioned on admission white blood cell count, C-reactive protein, calcium, and creatinine. These acute-phase measurements may mark early disease severity or lie on pathways to organ dysfunction. The broadly similar estimates across models support the stability of the observed conditional association, but adjustment for acute-phase variables cannot establish an effect of pre-illness insulin resistance or exclude reverse causation.

All fasting venous samples used to calculate METS-IR were obtained before acute treatment, and patients sampled after treatment were excluded. This reduces the possibility that treatment administered before sampling altered the analyzed glucose and lipid values; insulin-based regimens used in HTG-AP are known to lower triglycerides during hospitalization (28). Pretreatment sampling does not remove metabolic changes caused by pancreatitis itself. Reviews and cohort studies associate stress hyperglycemia with adverse AP outcomes (29–31). Differences in fasting duration and in the interval from symptom onset to sampling may also affect admission measurements. The persistence of an association after excluding patients with known diabetes therefore does not distinguish chronic insulin resistance from acute stress responses. Incomplete discharge lipid data prevented a paired admission-to-discharge METS-IR analysis, so these two sources of variation remain unresolved.

According to the reviewed medication histories, included patients had not received glucose-lowering or lipid-lowering treatment before admission. This limits one source of medication-related variation within our cohort, but the findings may not directly apply to treated populations. In a large real-world study of people with obesity or type 2 diabetes, initiation of a glucagon-like peptide-1 receptor agonist was associated with lower all-cause mortality and a small increase in acute pancreatitis risk, most evident early after initiation; no increased risk of chronic pancreatitis or pancreatic cancer was detected (32). That study did not evaluate METS-IR or HTG-AP and provides no explanation for our results. It instead illustrates why pancreatic outcomes in patients receiving metabolic treatment should be interpreted within the broader benefits and risks of that treatment.

This study has several limitations. First, its retrospective single-center design precludes causal inference, and residual confounding and limited generalizability remain. Second, METS-IR was calculated during an acute inflammatory illness. Although pretreatment sampling was verified, exact sampling-to-treatment intervals could not be consistently reconstructed. Discharge lipid measurements were not routinely available for all participants, preventing a paired discharge METS-IR analysis and leaving acute stress vs. underlying metabolic status unresolved. Third, the 411 screened records lacking valid METS-IR inputs were excluded. Variable-specific missingness could not be reliably reconstructed and no imputation-based sensitivity analysis was performed, so complete-case selection may have biased the association in an unknown direction. Finally, the ROC analyses show only apparent discrimination in this cohort. The data required to calculate APACHE II and CTSI were unavailable in the source records, so direct comparisons with these scores could not be performed. We did not assess calibration, incremental value beyond BISAP, or internal or external validation. METS-IR should therefore be interpreted as a pretreatment admission metabolic marker associated with SAP, rather than a validated clinical risk tool.

## 5 Conclusion

Among patients with HTG-AP, higher admission METS-IR was independently associated with higher odds of SAP. Restricted cubic spline analysis showed a predominantly increasing exposure-response relationship across the observed METS-IR range, without statistically significant evidence of nonlinearity. Although METS-IR may provide a simple and readily available marker of the metabolic phenotype associated with severe disease, its role in clinical assessment and generalizability remains uncertain. Prospective multicenter studies with external validation are required before METS-IR can be incorporated into routine risk stratification for HTG-AP.

## Data Availability

The raw data supporting the conclusions of this article will be made available by the authors, without undue reservation.

## Supplementary material

Supplementary Figure 1 Sequential adjustment of the METS-IR association.

Supplementary Figure 2 Exploratory ROC comparison of METS-IR and TyG.

Supplementary Figure 3 METS-IR association across the three-category outcome.

Supplementary Figure 4 LASSO cross-validation curve.

Supplementary Figure 5 LASSO coefficient paths.
